# Awareness and Interest in Rhinoplasty and Its Postoperative Complications Among Females in the Northern Border Region, Saudi Arabia

**DOI:** 10.7759/cureus.61209

**Published:** 2024-05-28

**Authors:** Yahia Abdelgawad Elsayed Elboraei, Rayan Alhumaidi R Alruwaili, Mohanad Talal k ALanazi, Yaser Yasen M Alnaser, Talal Maged Alenezi, Hamza A Alandijani, Mahdi Saleh Alanazi, Nawaf Lafi Ghayyadh Alruwaili, Safya E Esmaeel, Mohammad H Hussein

**Affiliations:** 1 Ear, Nose and Throat, College of Medicine, Northern Border University, Arar, SAU; 2 Medicine, College of Medicine, Northern Border University, Arar, SAU; 3 Otolaryngology, National Guard Hospital, Medina, Medina, SAU; 4 Physiology, College of Medicine, Northern Border University, Arar, SAU; 5 Surgery, Division of Endocrine and Oncologic Surgery, School of Medicine, Tulane University, New Orleans, USA

**Keywords:** saudi arabia, northern border region, interest, postoperative complications, awareness, rhinoplasty

## Abstract

Background: Rhinoplasty is a popular cosmetic surgical procedure that aims to reshape the nose, enhance its appearance, and improve nasal function. This study investigated the awareness, attitudes, and interest in rhinoplasty among females in the Northern Border Region of Saudi Arabia, as well as their knowledge of potential postoperative complications.

Methods: An online survey was used to perform a cross-sectional study. Female participants aged between 18 and 45 years, living in the Northern Border Region of Saudi Arabia, were selected. The questionnaire consisted of three main sections: personal information, attitude toward rhinoplasty, and complications of rhinoplasty.

Results: 905 females participated in our study. The majority (87.8%, n=795) had heard about rhinoplasty before, and 54.9% (n=497) knew someone who had undergone the surgery. Social media was the most common source of information about rhinoplasty (67.2%, n=608). A significant proportion of participants (72.4%, n=655) believed that their nose appearance sometimes or always limited their social and professional activities. However, only 16.7% (n=151) expressed a desire to change or improve their nose appearance through surgery. The educational status of the participant (*p*=0.027) and their father (*p*=0.011) were significantly associated with interest in rhinoplasty. Satisfaction with nose appearance, breathing, and family and friends' opinions about the participant's nose were also significantly associated with interest in rhinoplasty (*p*<0.001 for all). The majority of participants (88.4%, n=800) were aware of at least one complication, with the most recognized complications being breath disorders (74.6%, n=675), headache (70.6%, n=639), and mismatch of their new noses with the rest of their faces (69.8%, n=632). Age (*p*=0.008), city of residence (*p*<0.001), and satisfaction of family and friends with the participant's nose (*p*=0.019) were significantly associated with complication awareness.

Conclusion: This study found that women in Saudi Arabia's Northern Border Region had a high level of awareness and interest in rhinoplasty, despite concerns regarding the safety, availability of educational resources, and ethical considerations in promoting the procedure. The findings highlight the need for accurate and comprehensive information about rhinoplasty and its potential complications to be readily available to the public, particularly through targeted educational interventions and responsible advertising regulations.

## Introduction

The nose as a prominent facial feature plays a significant role in determining an individual's overall facial aesthetic and beauty. It is intrinsically linked to self-worth, self-confidence, and self-image of an individual. Any changes to the nasal structure, whether brought about by surgery, trauma, or inherited conditions, can significantly affect a person's mental health [1].

Rhinoplasty, a popular cosmetic surgical procedure, aims to reshape the nose, enhance its appearance, and improve nasal function. Individuals dissatisfied with their nose's appearance may opt for rhinoplasty to alter its shape or size while maintaining or enhancing nasal airway function. As a cosmetic procedure, rhinoplasty and its revision surgeries can have significant aesthetic and psychological effects on the patient [2].

However, like any surgical intervention, rhinoplasty carries inherent risks and potential complications. Early effects include epistaxis, periorbital ecchymosis, septal hematoma, infection, and skin necrosis; late repercussions include scar hypertrophy, septal perforation, and enophthalmos [3]. While serious problems are uncommon, a range of short- and long-term issues can cause patient discomfort, cosmetic dissatisfaction, and even functional difficulties [4].

In the last few years, the global demand for rhinoplasty has experienced a notable increase, partially attributed to the rising prevalence of body dysmorphic disorders [5]. In Saudi Arabia, the demand for rhinoplasty is particularly high, underscoring the importance of assessing the awareness and interest in this procedure among the female population. Moreover, understanding their knowledge of potential postoperative complications is crucial for promoting informed decision-making and ensuring patient safety.

The primary objectives of this study are to evaluate the awareness and level of interest in rhinoplasty among females in the Northern Border Region of Saudi Arabia and to assess their knowledge of the procedure's potential postoperative consequences. By examining these factors, the study aims to provide valuable insights into the current state of knowledge and attitudes towards rhinoplasty in this population. The findings of this research can inform future educational initiatives and patient counselling strategies, ultimately contributing to informed decision-making and optimizing patient outcomes in the context of rhinoplasty.

## Materials and methods

Study design and participants

A cross-sectional study was conducted using a quantitative approach. Female participants aged between 18 and 45 years, living in the Northern Border Region of Saudi Arabia, were selected through convenience sampling. Inclusion criteria were agreeing to complete the survey and having an interest in rhinoplasty, while exclusion criteria were refusing to participate.

The study prioritized participants' confidentiality and data privacy. Deidentified patient information was used, and each participant was assigned a unique code to maintain anonymity. Ethical clearance was obtained from the Ethical Committee of the College of Medicine (HAP-09-A-043) and was issued by decision no (19-24-H) at Northern Border University before the commencement of the study.

After participants read and approved the informed consent, an online questionnaire, produced in Arabic, was made available via an anonymous online survey tool [6].

The questionnaire consisted of three main sections.

Personal Information

This section included questions on age, nationality, marital status, educational status, educational status of parents, and city of residence.

Attitude towards Rhinoplasty

Participants were asked about their satisfaction with their nose appearance and breathing, the impact of their nose on social and professional activities, their desire to change or improve their nose through surgery, and their familiarity with rhinoplasty. Additional questions explored the sources of information about rhinoplasty, the influence of media on perceptions and decisions related to the procedure, and the availability of educational resources.

Complications of Rhinoplasty

Participants were asked about their familiarity with various postoperative complications, such as skin discolouration, breath disorders, recurrent nosebleeds, nose blockage, recurrent nasal mucosal irritation, headache, recurrent nausea and vomiting, nasal discharge, sensitivity to strong odours, death, need for reoperation, dissatisfaction with the new nose, and mismatch of the new nose with the rest of the face.

Statistical analysis

A sample size of 905 participants was estimated using the Raosoft® calculator, with a 5% level of significance, 5% margin of error, 95% confidence, and expected response distribution of 50%. Data analysis was performed using SPSS (version 28) and RStudio 2023.06.0 Build 421. Descriptive statistics clarify the participants' sociodemographic characteristics, their attitudes towards rhinoplasty, and their awareness of postoperative complications. Continuous variables were expressed as mean ± standard deviation, while categorical variables were presented as frequencies and percentages. The Chi-square test assessed the relationships between categorical variables, such as sociodemographic factors, interest in rhinoplasty, and awareness of postoperative complications. Logistic regression analysis was performed to identify the factors associated with interest in rhinoplasty and awareness of postoperative complications. Odds ratios (ORs) and their corresponding 95% confidence intervals (CIs) were calculated to measure the strength of the associations. A p-value of less than 0.05 was considered statistically significant.

For questions using Likert scales (e.g., satisfaction levels), descriptive statistics such as frequencies, percentages, and modes were calculated to show the responses. The mean score for each Likert scale question was also calculated to provide an overall measure of satisfaction or agreement. For open-ended questions (e.g., reasons for wanting to change/improve the nose), thematic analysis was conducted to identify common themes and patterns in the responses. The survey responses were compared between different subgroups, such as age groups, educational levels, or cities, to identify any significant differences in attitudes, knowledge, or preferences related to rhinoplasty. Independent t-tests or one-way ANOVA were used for comparing continuous variables, while chi-square tests were used for categorical variables.

## Results

A total of 905 individuals participated in the survey. Most of the participants were aged between 26 and 35 years (39.8%, n=360), followed by those over 40 years old (32.6%, n=295). Nearly half of the participants were single (48.1%, n=435), and 45.5% (n= 412) were married. Most of the participants had a university education or higher (78.7%, n=712). Most of the participants were Saudi nationals (95.4%, n=863), and 88.5%(n=801) were from the city of Arar (Table 1).

**Table 1:** Sociodemographic characteristics of the participants (n=905) Data has been represented as N,%.

Characteristic	Category	Frequency (N)	Percentage (%)
Age (years)	18-25	48	5.3
	26-35	360	39.8
	36-40	202	22.3
	More than 40	295	32.6
Marital status	Married	412	45.5
	Single	435	48.1
	Divorced	42	4.6
	Widowed	16	1.8
Educational status	Illiterate	4	0.4
	Primary school	5	0.6
	Preparatory school	32	3.5
	Secondary school	152	16.8
	University or higher	712	78.7
Nationality	Non-Saudi	42	4.6
	Saudi	863	95.4
City of residence	Arar	801	88.5
	Turaif	35	3.9
	Rafha	26	2.9
	Other	43	4.8

The participants reported varying levels of satisfaction with their nose appearance and function. More than one-third of the participants (36.9%, n=334) were completely satisfied with their nose appearance, while 39.4% (n=357)were completely satisfied with their nose breathing. A similar proportion (40.2%, n=364) reported that their family and friends were completely satisfied with the appearance of their noses (Table 2). Likert scale analysis further supported these findings, with mean scores of 3.86 ± 1.13 for satisfaction with nose appearance, 3.89 ± 1.18 for satisfaction with nose breathing, and 3.96 ± 1.09 for satisfaction of family and friends with the participant's nose appearance. These results suggest generally high levels of satisfaction across all three domains.

**Table 2:** Satisfaction levels with nose appearance and function (n=905) Data has been represented as N,%.

Question	Unsatisfied	Poorly satisfied	Moderately satisfied	Very satisfied	Completely satisfied
How do you feel about your nose?	50 (5.5)	43 (4.8)	224 (24.8)	254 (28.1)	334 (36.9)
How satisfied are you with your nose breathing?	57 (6.3)	49 (5.4)	188 (20.8)	254 (28.1)	357 (39.4)
How satisfied are your family and friends with the appearance of your nose?	42 (4.6)	33 (3.6)	212 (23.4)	254 (28.1)	364 (40.2)

**Figure 1:**
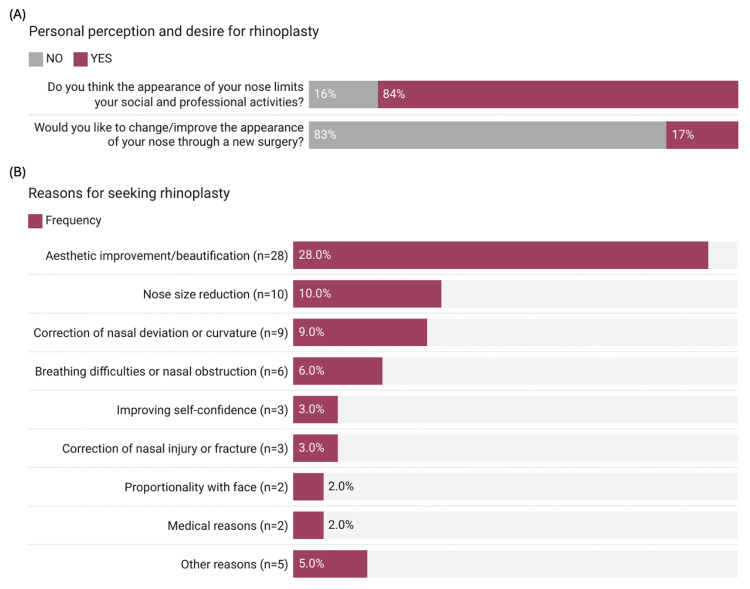
Perception and impact of rhinoplasty on activities (A) Personal perception and desire for rhinoplasty (n=905) Data has been represented as %; (B) Reasons for seeking rhinoplasty (n=68). Data has been represented as N,%.

A significant proportion of the participants (72.4%, n=655) believed that their nose appearance sometimes (11.6%, n=105) or always (60.8%, n=576) limited their social and professional activities. However, despite this perception, only 16.7% (n=151) expressed a desire to change or improve their nose appearance through surgery, while the majority (83.3%, n=754) did not wish to undergo rhinoplasty (Figure 1A). Among the participants who provided reasons for considering rhinoplasty (n=68) as 7.4% from total, the most common reason was aesthetic improvement or beautification (41.2%, n=28), followed by nose size reduction (14.7%, n=10), correction of nasal deviation or curvature (13.2%, n=9), and breathing difficulties or nasal obstruction (8.8%, n=6). Other reasons included improving self-confidence (4.4%, n=3), correction of nasal injury or fracture (4.4%, n=3), proportionality with face (2.9%, n=2), medical reasons (2.9%, n=2), and other reasons (7.4%, n=5) (Figure 1B).

The study compared the characteristics of participants who were interested and not interested in rhinoplasty (Table 3). The results indicated that the educational status of the participant (p=0.027) and their father (p=0.011) were significantly associated with interest in rhinoplasty, with those having lower educational levels being more interested. Satisfaction with nose appearance, breathing, and family and friends' opinions about the participant's nose were also significantly associated with interest in rhinoplasty (p<0.001 for all), with lower satisfaction levels (indicated by lower Likert scale scores) being associated with higher interest.

**Table 3:** Association between socioeconomic factors and interest in rhinoplasty Data has been represented as N,% or Mean ± SD. Two-sided Chi-Square or independent t-tests were employed. P value is considered significant (p < 0.05%, p < 0.001%).

Characteristic	Category	Not interested (n=754)	Interested (n=151)	p-value
Age (years)	18-25	34 (4.5)	14 (9.3)	0.06
	26-35	296 (39.3)	64 (42.4)	
	36-40	169 (22.4)	33 (21.9)	
	More than 40	255 (33.8)	40 (26.5)	
Marital status	Married	344 (45.6)	68 (45)	0.13
	Single	368 (48.8)	67 (44.4)	
	Divorced	31 (4.1)	11 (7.3)	
	Widowed	11 (1.5)	5 (3.3)	
Participant's educational level	Illiterate/Primary/Preparatory	29 (3.8)	12 (7.9)	0.027
	Secondary school/University	725 (96.2)	139 (92.1)	
Father's educational level	Illiterate/Primary/Preparatory	210 (27.9)	27 (17.9)	0.011
	Secondary school/University	544 (72.1)	124 (82.1)	
Mother's educational level	Illiterate/Primary/Preparatory	247 (32.8)	49 (32.5)	0.94
	Secondary school/University	507 (67.2)	102 (67.5)	
Nationality	Non-Saudi	33 (4.4)	9 (6)	0.40
	Saudi	721 (95.6)	142 (94)	
City of residence	Arar	666 (88.3)	135 (89.4)	0.64
	Turaif	31 (4.1)	4 (2.6)	
	Rafha	20 (2.7)	6 (4)	
	Other	37 (4.9)	6 (4)	
Satisfaction level	Satisfaction with nose appearance	3.1 ± 0.9	1.8 ± 1.4	<0.001
	Satisfaction with nose breathing	3.1 ± 1.0	1.9 ± 1.5	<0.001
	Family/friends' satisfaction with nose appearance	3.2 ± 0.9	1.9 ± 1.4	<0.001

**Figure 2:**
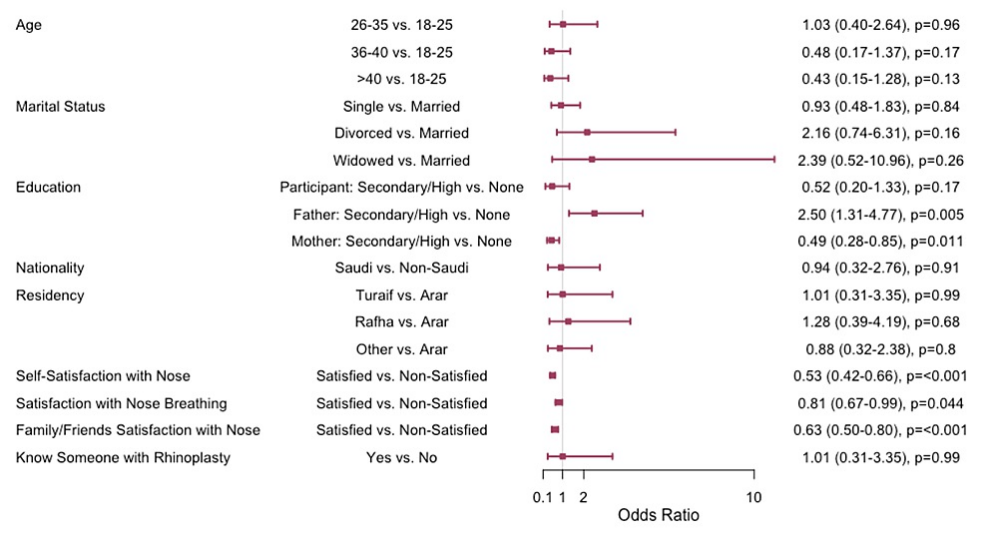
Factors associated with the desire to undergo rhinoplasty Logistic regression analysis was performed. Data has been represented as odds ratio (OR) and 95% confidence intervals (CI). P value is considered significant (p < 0.05%, p < 0.001%).

Logistic regression analysis (Figure 2) revealed that participants who were satisfied with their nose appearance (OR=0.53, 95% CI: 0.42-0.66, p<0.001), satisfied with their nose breathing (OR=0.81, 95% CI: 0.67-0.99, p=0.044), and whose family and friends were satisfied with their nose appearance (OR=0.63, 95% CI: 0.50-0.80, p<0.001) were less likely to be interested in rhinoplasty. The educational status of the participant's father was a significant predictor, with those whose fathers had secondary school or higher education being more likely to be interested in rhinoplasty compared to those whose fathers had low education (OR=2.50, 95% CI: 1.31-4.77, p=0.005). Conversely, participants whose mothers had secondary school or higher education were less likely to be interested in rhinoplasty compared to those whose mothers had no formal education (OR=0.49, 95% CI: 0.28-0.85, p=0.011). Age, marital status, nationality, and city of residence were not significantly associated with interest in rhinoplasty. Knowing someone who had undergone rhinoplasty was not significantly associated with interest in the procedure.

The majority of the participants (87.8%, n=795) had heard about rhinoplasty before, and more than half (54.9%, n=497) knew someone who had undergone the surgery. Social media was the most common source of information about rhinoplasty (67.2%, n=608), followed by friends and family (14.8%, n=134), television (8.0%, n=72), and Internet searches (5.9%, n=53). The frequency of encountering rhinoplasty news on social media varied, with 30.1% (n=272) reporting rare encounters and 13.0% (n=118) reporting daily encounters (Table 4).

**Table 4:** Awareness of rhinoplasty and sources of information (n=905) Data has been represented as N,%.

Characteristic	Category	Frequency (N)	Percentage (%)
Awareness of rhinoplasty	Heard about rhinoplasty before	795	87.8
	Know anyone who has had rhinoplasty surgery	497	54.9
Sources of information about rhinoplasty	Social media	608	67.2
	Friends/Family	134	14.8
	Television	72	8.0
	Internet search	53	5.9
	Other	38	4.2
Frequency of rhinoplasty news on social media	Never	73	8.1
	Rarely	272	30.1
	Monthly	256	28.3
	Weekly	186	20.6
	Daily	118	13.0

**Figure 3:**
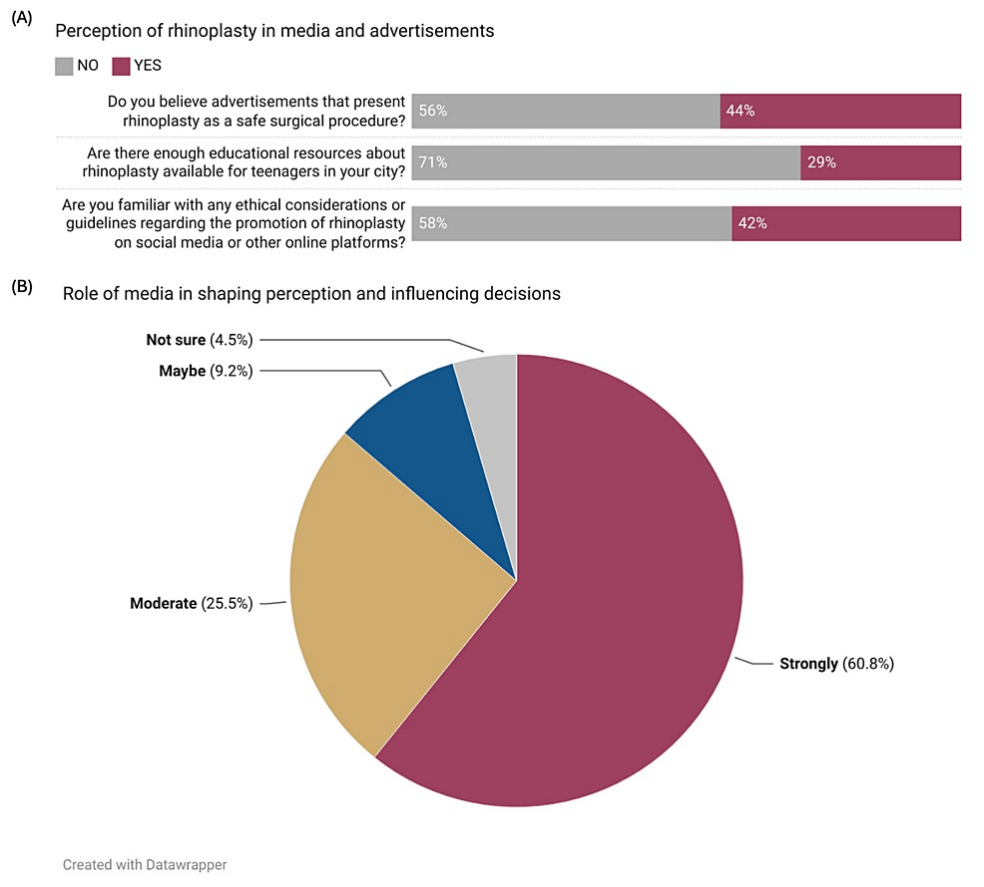
Perception of rhinoplasty in media and the role of media in shaping decisions (n=905) (A) Perception of rhinoplasty in media and advertisements; (B) Role of media in shaping perception and influencing decisions. Data has been represented as %.

Despite the high awareness of rhinoplasty, more than half of the participants (56.0%, n=507) did not believe advertisements presenting rhinoplasty as a safe surgical procedure. The majority (70.8%, n=641) also felt that there were insufficient educational resources about rhinoplasty available for teenagers in their city. Furthermore, 58.2% (n=527) of the participants were not familiar with any ethical considerations or guidelines regarding the promotion of rhinoplasty on social media or other online platforms (Figure 3A). Most participants strongly believed (60.8%, n=550) or moderately believed (25.5%, n=231) that media plays a role in shaping perception and influencing decisions related to rhinoplasty among teenagers, while a smaller proportion were unsure (4.5%, n=41) or thought media might play a role (9.2%, n=83) (Figure 3B). Likert scale analysis further reinforced these findings, with a mean score of 3.38 ± 0.87 for the perceived role of media in shaping perception and influencing decisions related to rhinoplasty among teenagers, suggesting a strong belief in the media's influence. Logistic regression analysis showed that belief in the effect of media on the desire to undergo rhinoplasty was significantly associated with an increased likelihood of being interested in the procedure (OR=1.44, 95% CI: 1.13-1.86, p=0.004).

Most participants (79.9%, n=723) believed that more stringent regulations should be on advertising and promoting rhinoplasty services targeting teenagers (Table 5). Regarding comfort levels in discussing rhinoplasty with friends or family, 28.3% (n=256) reported feeling very comfortable, while 13.8% (n=125) felt uncomfortable (Table 5).

**Table 5:** Regulations and comfort level discussing rhinoplasty (n=905) Data has been represented as N,%.

Characteristic	Category	Frequency (N)	Percentage(%)
Should there be more stringent regulations on the advertisement and promotion of rhinoplasty services targeting teenagers?	No	182	20.1
	Yes	723	79.9
How comfortable do you feel discussing the topic of rhinoplasty with your friends, or family?	Very comfortable	256	28.3
	Moderately comfortable	272	30.1
	Slightly comfortable	147	16.2
	Uncomfortable	125	13.8
	Prefer not to answer	105	11.6

**Figure 4:**
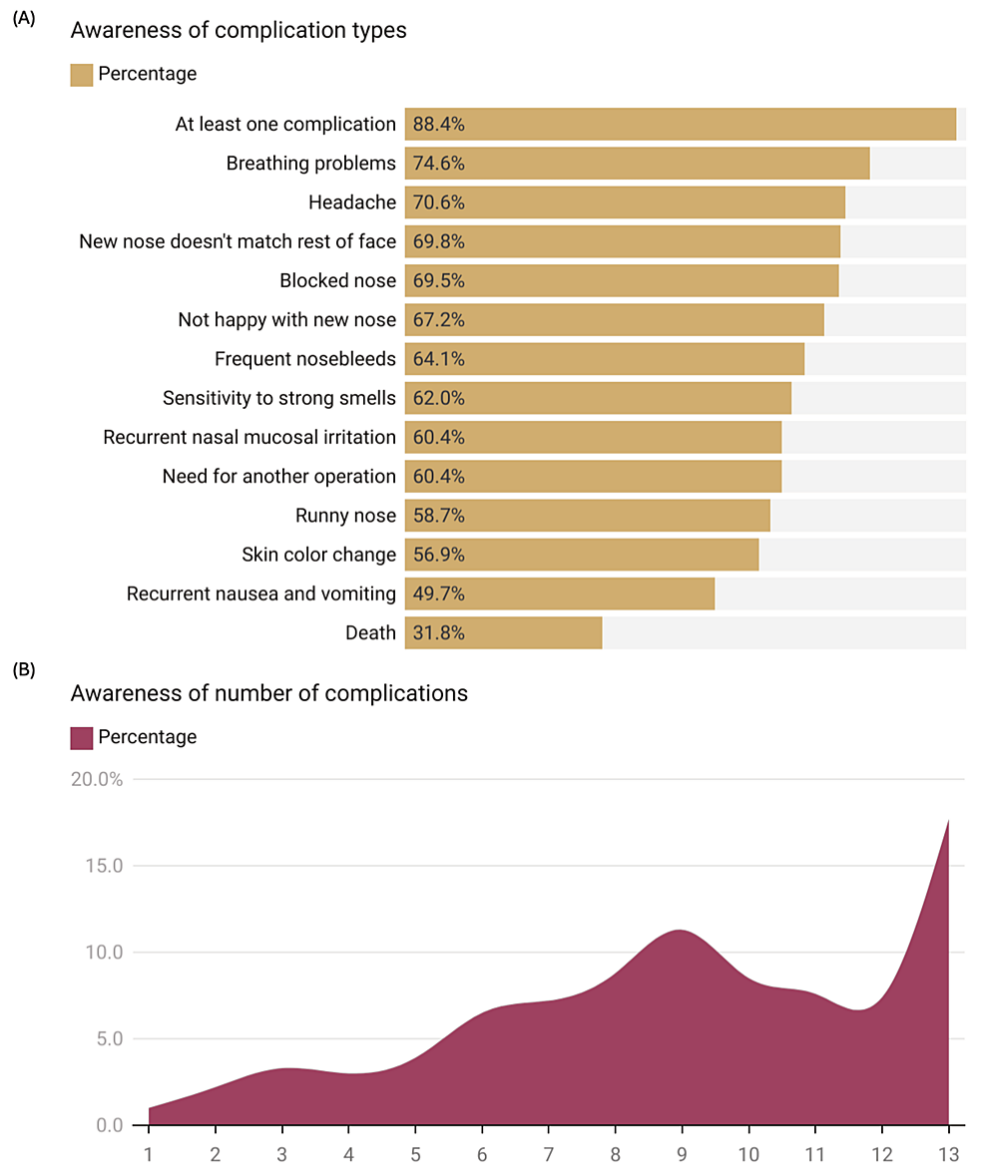
Awareness of postoperative complications related to rhinoplasty (A) Knowledge of complication types after rhinoplasty (n=905). A bar graph showing the percentage of participants aware of each specific postoperative complication related to rhinoplasty; (B) Distribution of participants by number of postoperative complications they were aware of (n=905). A histogram displaying the distribution of participants according to the number of postoperative complications they were aware of, ranging from 1 to 13. Data has been represented as %.

The study investigated participants' awareness of postoperative complications related to rhinoplasty. The results showed that 88.4% (n=800) of participants were aware of at least one complication. The most recognized complications were breath disorders (74.6%, n=675), headache (70.6%, n=639), and mismatch of the new nose with the rest of the face (69.8%, n=632). The least recognized complications were recurrent nausea and vomiting (49.7%, n=450) and the possibility of death (31.8%, n=288) (Figure 4A). The number of complications participants were aware of varied considerably. About 17.7% (n=160) of participants knew about all 13 complications listed in the survey, while 11.6% (n=105) were unaware of any complications. The remaining participants demonstrated awareness of between 1 and 12 complications (Figure 4B).

Age (p=0.008), city of residence (p < 0.001), and satisfaction of family and friends with the participant's nose (p=0.019) were significantly associated with complication awareness. Middle-aged participants (26-40 years) and those living in Arar were more likely to know about potential problems after surgery, while those whose loved ones were less satisfied with their nose appearance were more aware of complications (Table 6).

**Table 6:** Association between sociodemographic factors and awareness of postoperative complications Data has been represented as N,% or Mean ± SD. Two-sided Chi-Square or independent t-tests were employed. P value is considered significant (p < 0.05%, p < 0.001%).

Characteristic	Category	Not aware (n=105)	Aware (n=800)	p-value
Age (years)	18-25	10 (9.5)	38 (4.8)	0.008
	26-35	33 (31.4)	327 (40.9)	
	36-40	17 (16.2)	185 (23.1)	
	More than 40	45 (42.9)	250 (31.3)	
Marital status	Married	41 (39)	371 (46.4)	0.35
	Single	56 (53.3)	379 (47.4)	
	Divorced	7 (6.7)	35 (4.4)	
	Widowed	1 (1)	15 (1.9)	
Educational status of the participant	Illiterate/Primary/Preparatory	4 (3.8)	37 (4.6)	0.71
	Secondary school/University	101 (96.2)	763 (95.4)	
The educational status of the father	Illiterate/Primary/Preparatory	26 (24.8)	211 (26.4)	0.81
	Secondary school/University	79 (75.2)	589 (73.6)	
Educational status of the mother	Illiterate/Primary/Preparatory	31 (29.5)	265 (33.1)	0.51
	Secondary school/University	74 (70.5)	535 (66.9)	
Nationality	Non-Saudi	4 (3.8)	38 (4.8)	0.81
	Saudi	101 (96.2)	762 (95.3)	
City	Arar	81 (77.1)	720 (90)	<0.001
	Turaif	9 (8.6)	26 (3.3)	
	Rafha	3 (2.9)	23 (2.9)	
	Other	12 (11.4)	31 (3.9)	
Self-satisfaction with nose	Mean ± SD	3.0 ± 1.1	2.8 ± 1.1	0.11
Satisfaction with nose breathing	Mean ± SD	2.9 ± 1.2	2.8 ± 1.2	0.45
Family/friends satisfied with your nose	Mean ± SD	3.2 ± 0.97	2.92 ± 1.1	0.019

**Figure 5:**
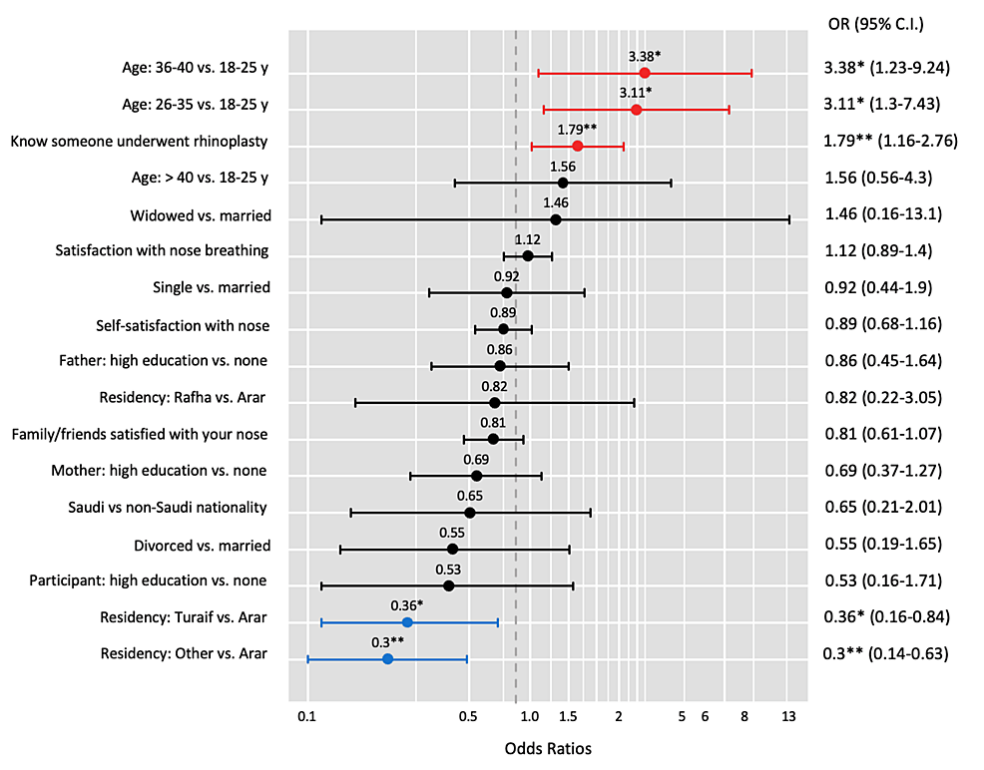
Predictors of awareness of complications Multivariate logistic regression analysis was performed, and results are reported as odds ratio (OR) and 95% confidence interval (CI). High education: Secondary school or university education

Figure 5 presents the results of a logistic regression analysis examining the predictors of awareness of postoperative complications related to rhinoplasty. Age was a significant predictor of complication awareness, with participants aged 26-35 years (OR=3.11, 95% CI: 1.30-7.43, p=0.011) and 36-40 years (OR=3.38, 95% CI: 1.23-9.24, p=0.018) being more likely to be aware of complications compared to those aged 18-25 years. Participants who knew someone with a history of rhinoplasty were more likely to be aware of potential postoperative complications (OR=1.79, 95% CI: 1.16-2.76, p=0.009). Residency was a significant predictor, with participants living in Turaif (OR=0.36, 95% CI: 0.16-0.84, p=0.017) and other cities (OR=0.30, 95% CI: 0.14-0.63, p=0.002) being less likely to be aware of complications compared to those living in Arar. Marital status, educational status of the participant and their parents, and nationality were not significantly associated with complication awareness.

## Discussion

The present study aimed to investigate the awareness, attitudes, and interest in rhinoplasty among females in the Northern Border Region of Saudi Arabia, as well as their knowledge of potential postoperative complications. The findings provide valuable insights into the complex factors influencing the perception and decision-making process regarding this cosmetic procedure.

One of the study's main conclusions was that participants had a high level of awareness about rhinoplasty, with 87.8% having heard of the procedure previously and 54.9% knowing someone who had had the surgery. This awareness has increased with the use of septorhinoplasty because of advancements in surgical technique, media awareness, and improved patient interest [7]. Moreover, a recent study suggested that some people who are dissatisfied with their noses may have rhinoplasty to alter the shape of the nose [8]. Both men and women in the younger age group often exhibit this tendency [9]. This high awareness can be attributed to the increasing popularity of rhinoplasty in Saudi Arabia and the significant role played by social media in disseminating information about the procedure. Social media was identified as the most common source of information about rhinoplasty (67.2%), followed by friends and family (14.8%) consistent with social media significantly affecting patients’ decisions [10]. In addition, another article suggested that social media has an important role in explaining the increased desire for cosmetic procedures [11]. This finding underscores the importance of social media platforms in shaping public perception and influencing decisions related to cosmetic surgery.

Despite the high awareness, the study revealed that a significant proportion of participants (72.4%) believed that their nose appearance sometimes or always limited their social and professional activities, these findings were consistent with another study that revealed that rhinoplasty has unique preoperative and postoperative psychological factors [12]. This finding suggests that perceived physical imperfections can affect an individual's self-image and confidence, potentially affecting their personal and professional life. However, it is noteworthy that only 16.7% of the participants desired to change or improve their nose appearance through surgery. In contrast, another Saudi study revealed that more than half of the participants didn’t accept cosmetic surgery [13]. This discrepancy highlights the complex attitudes toward cosmetic surgery and the fact that not all individuals who are dissatisfied with their appearance may choose to undergo surgical intervention.

The study also identified several sociodemographic factors associated with interest in rhinoplasty. The educational status of the participants and their fathers were found to be significantly associated with interest in the procedure, with those of lower educational levels being more interested, which aligns with a previous study that has proposed that the rate of cosmetic surgery falls as parental education rises [14]. Nonetheless, a recent study found no connection between parental education and rhinoplasty interest [15]. This finding suggests that individuals with lower educational backgrounds may be more susceptible to media influences and may view rhinoplasty as a means to improve their social status and enhance their prospects in various aspects of life.

Additionally, satisfaction with nose appearance, breathing, and family and friends' opinions about the participant's nose were significantly associated with interest in rhinoplasty, with lower satisfaction levels being associated with higher interest [8]. This highlights the role of personal and social factors in shaping the desire for cosmetic surgery.

Another important finding of the study was the participants' concern regarding the safety, availability of educational resources, and ethical considerations in the promotion of rhinoplasty, particularly on social media platforms. The majority of participants did not believe advertisements presenting rhinoplasty as a safe procedure (56.0%), felt there were insufficient educational resources about rhinoplasty available for teenagers in their city (70.8%), and were not familiar with ethical considerations or guidelines regarding the promotion of rhinoplasty on social media or other online platforms (58.2%). These findings underscore the need for accurate and comprehensive information about the risks, benefits, and long-term consequences of rhinoplasty to be made readily available to the public, especially teenagers who may be more vulnerable to media influences.

The study also investigated participants' awareness of postoperative complications related to rhinoplasty. While 88.4% of participants were aware of at least one complication, the level of awareness varied considerably across different complications in contrast with the previous study which revealed that More than half of the participants in our study possessed below-average information regarding the complications that arise after rhinoplastic surgery [3,16]. The most recognized complications were breath disorders (74.6%), and headache (70.6%). In line with another study, the most common rhinoplasty problems identified by the respondents were headache, respiratory issues, and unhappiness with the new nose (69.8%). mismatch of the new nose with the rest of the face [15]. While the least recognized complications were recurrent nausea and vomiting (49.7%) and the possibility of death (31.8%). These findings align with life-threatening complications that occur in 1.7% to 5% of rhinoplasty [17]. This variation in awareness highlights the need for more comprehensive education about the full range of potential complications associated with rhinoplasty.

The study identified several factors associated with complication awareness, including age, city of residence, and family and friends ‘ satisfaction with the participant's nose. Middle-aged participants (26-40 years) and those living in Arar were more likely to be aware of potential problems after surgery. Moreover, those whose loved ones were less satisfied with their nose appearance were more aware of complications. Studies have revealed that teenage females have less knowledge about rhinoplasty and its complications [6,17]. In another study in California, 30% of participants were influenced by someone who had undergone the surgery [18].

The logistic regression analysis revealed that age, knowing someone who had undergone rhinoplasty, and residency were significant predictors of complication awareness. Participants aged 26-35 years and 36-40 years were more likely to be aware of complications compared to those aged 18-25 years, highlighting the importance of age and life experience in shaping knowledge about the risks associated with cosmetic surgery. Participants who knew someone with a history of rhinoplasty were also more likely to be aware of potential postoperative complications, suggesting that personal connections and experiences of others can influence an individual's understanding of the risks involved. Additionally, participants living in Turaif and other cities were less likely to be aware of complications compared to those living in Arar, these findings align with another study that showed that there were potential geographic disparities in access to information and education about rhinoplasty [19].

The findings of this study have important implications for healthcare professionals, policymakers, and society as a whole. The highest level of awareness and interest in rhinoplasty among females in the Northern Border Region of Saudi Arabia underscores the need for accurate and comprehensive information about the procedure to be available to the public. The findings were similar to another study that showed a high awareness of rhinoplasty among females and a high interest in cosmetic surgery [20].

Healthcare professionals should focus on developing targeted educational interventions to improve patient knowledge and informed decision-making, particularly among younger individuals, those with lower education levels, and those residing in areas outside of Arar. These interventions should aim to increase awareness of the potential complications associated with rhinoplasty and provide guidance on critically evaluating information presented on social media and other online platforms. Influencers who have had rhinoplasty surgery falsely advertised the process on social media, which made the public less aware of the risks associated with the practice [21].

So, policymakers should also consider implementing regulations to ensure that advertisements and promotions of rhinoplasty services, particularly those targeting teenagers, are accurate, transparent, and ethically responsible. This may involve developing guidelines for promoting cosmetic surgery on social media platforms and increasing oversight of the content presented in these advertisements.

Additionally, society should engage in open and honest conversations about the societal pressures and beauty standards that may contribute to the increasing popularity of cosmetic surgery, particularly among younger individuals. By fostering a culture of self-acceptance and body positivity, we can help individuals develop a more positive self-image and reduce the perceived need for surgical intervention to address physical imperfections.

The putative hypothesis for those who are less educated being more interested in rhinoplasty could be related to several factors:

Socioeconomic status

Lower educational levels are often associated with lower socioeconomic status. Individuals from lower socioeconomic backgrounds may view rhinoplasty as a means to improve their social status and enhance their prospects in various aspects of life, such as employment or relationships.

Exposure to media influences

Less educated individuals may be more susceptible to media influences, such as celebrity culture and advertising, which often promote idealized beauty standards. This increased exposure and vulnerability to media messages may lead to a greater desire for cosmetic enhancements, including rhinoplasty.

Limited access to information

Individuals with lower educational levels may have limited access to accurate and comprehensive information about the risks, benefits, and long-term consequences of rhinoplasty. This lack of information may result in a more idealized perception of the procedure and its outcomes, leading to a higher interest in undergoing rhinoplasty.

Psychological factors

Lower educational levels may be associated with lower self-esteem and body satisfaction. Individuals who are less satisfied with their appearance may view rhinoplasty as a means to boost their self-confidence and improve their overall psychological well-being.

Cultural and societal factors

In some cultures or societies, physical appearance may be more heavily emphasized and valued, particularly among those with lower educational levels. This cultural pressure to conform to certain beauty standards may contribute to a higher interest in cosmetic procedures like rhinoplasty.

This information can be valuable for healthcare professionals and policymakers in developing targeted educational interventions to improve patient knowledge and informed decision-making. These findings highlight the importance of considering sociodemographic factors when developing educational interventions to improve patient knowledge and informed decision-making regarding rhinoplasty. Healthcare professionals should focus on increasing awareness of potential complications, particularly among younger individuals, those with lower education levels, and those residing outside Arar. By tailoring educational efforts to these specific groups, healthcare providers can help ensure that patients are well-informed about the risks associated with rhinoplasty and can make more informed decisions about whether to undergo the procedure

This discrepancy highlights complex attitudes towards cosmetic surgery and the impact of perceived physical imperfections on personal and professional life.

It is important to take into account the many limitations of this study when evaluating the findings. First, the sample was restricted to Saudi Arabia's Northern Border Region's female population, which may not accurately reflect the country's overall population or those of other regions. Second, the research used self-reported data, which is prone to biases related to social desirability and recollection. Third, the cross-sectional form of the study makes it impossible to determine a causal relationship between the variables under investigation. Fourth, the study did not investigate the precise causes of the participants' desire for rhinoplasty.

## Conclusions

In conclusion, this study provides valuable insights into the awareness, attitudes, and interest in rhinoplasty among females in the Northern Border Region of Saudi Arabia, as well as their knowledge of potential postoperative complications. The findings highlight the complex factors influencing the perception and decision-making process regarding this cosmetic procedure and underscore the need for accurate and comprehensive information to be made readily available to the public. By developing targeted educational interventions, implementing responsible advertising regulations, and fostering a culture of self-acceptance, we can help individuals make informed decisions about whether to undergo rhinoplasty and ultimately promote better physical and mental health outcomes.
